# Environmental influences on African migration to Canada: focus group findings from Ottawa-Gatineau

**DOI:** 10.1007/s11111-014-0214-3

**Published:** 2014-07-12

**Authors:** Luisa Veronis, Robert McLeman

**Affiliations:** 1 Department of Geography, University of Ottawa, 60 University, Room 017, Ottawa, ON K1N 6N5 Canada; 2 Department of Geography and Environmental Studies, Wilfrid Laurier University, 75 University Avenue West, Waterloo, ON N2L 3C5 Canada

**Keywords:** International migration, Global South–North migration, Horn of Africa, Sub-Saharan Africa, Urban ecological decline, Migration motivations, Migration decisions

## Abstract

There is limited empirical evidence of how environmental conditions in the Global South may influence long-distance international migration to the Global North. This research note reports findings from seven focus groups held in Ottawa-Gatineau, Canada, with recent migrants from the Horn of Africa and francophone sub-Saharan Africa, where the role of environment in migration decision-making was discussed. Participants stated that those most affected by environmental challenges in their home countries lack the financial wherewithal to migrate to Canada. Participants also suggested that internal rural–urban migration patterns generated by environmental challenges in their home countries underlay socioeconomic factors that contributed to their own migration. In other words, environment is a second- or third-order contributor in a complex chain of interactions in the migrant source country that may lead to long-distance international migration by skilled and educated urbanites. These findings have informed the scope and detail of a larger, ongoing empirical study of environmental influences on immigration to Canada.

**Electronic supplementary material:**

The online version of this article (doi:10.1007/s11111-014-0214-3) contains supplementary material, which is available to authorized users.

## Introduction

Popular and scholarly discussions of environmental influences on long-distance international migration have often been informed by the “environmental refugee” paradigm (El-Hinnawi 1985), which suggests that declining environmental quality and climate change is driving large numbers of people, especially in the Global South, into short- and long-distance migration (see Bates 2002; Gill 2010 for reviews). In a study that continues to be widely cited, Myers (2002) suggested there could be as many as 200 million environmental refugees worldwide by mid-century, most originating in less developed countries. Subsequent research has shown that the relationship between environmental processes and human migration behavior is complex and dynamic and does not necessarily unfold in the stimulus–response fashion the environmental refugee paradigm suggests. Instead, environmental migration is increasingly seen as one of a range of possible ways by which vulnerable populations adjust and adapt to environmental risks and hazards (Hunter 2005; Tacoli 2009; McLeman and Smit 2006), or as a means by which people seek to take advantage of environmental amenities and opportunities (Rappaport 2007; Gutmann and Field 2010).

Researchers are now seeking to understand better the processes by which environmental events and conditions interact with cultural, demographic, economic, institutional, political, and social forces to influence migration decisions and behavior (Black et al. 2011; Foresight 2011). Empirical studies have shown that these interactions can have a direct and obvious influence on migration, such as migration that emerges in the wake of natural hazard events (e.g., Schultz and Elliott 2013) or a subtle or indirect one, such as gradual changes in migration flows that emerge as a result of long-term land degradation caused by human activities (e.g., Henry et al. 2004; Shrestha and Bhandari 2007). Further, it is now recognized that a given environmental event or condition might lead to a variety of different types of migration responses and other forms of adaptation, as was seen, for example, in the wake of Hurricane Katrina (Fussell et al. 2010; Groen and Polivka 2010). The available empirical evidence suggests that, in most instances, migration that occurs in response to environmental phenomena takes place internally within states or intra-regionally between contiguous countries, often following preexisting social networks (McLeman 2013). Examples include drought-related migration in dryland Africa and between Mexico and the USA (e.g., Barbier et al. 2009; Gray and Mueller 2012a; Nawrotzki et al. 2013), and flooding-related migration in Bangladesh and in Southeast Asia (e.g., Gray and Mueller 2012b; Dun 2011). Nonetheless, there continues to be a preoccupation in the popular media and among global policymakers with the potential for greater levels of international environmental migration, especially from the Global South. Calls are emerging from within the academy and from governments to use the United Nations Framework Convention on Climate Change (UNFCCC) process to create policies and programs to protect those who may be displaced or seek to migrate internationally because of climate change (Biermann and Boas 2012; Gibb and Ford 2012).

This attention to long-distance climate-related migration reflects valid concerns that populations in less developed countries are especially vulnerable to the adverse future impacts of anthropogenic climate change and mean sea level rise (Füssel 2010). Further, general equilibrium models developed by Marchiori and Schumacher (2011) suggest that environmentally related international migration could indeed rise as a result of climate change. However, an important challenge faced by scholars and policymakers alike is that empirical evidence on the influence of environment on long-distance international migration from the Global South to the Global North is quite limited and typically embedded within larger studies of environmental migration patterns from particular countries. Examples include studies of migration from Niger and Mali to Europe (Findley 1994; Afifi 2011), the Dominican Republic to the USA (Alscher 2011), Ecuador to the USA and Europe (Gray 2009, 2010), and Honduras to the USA (Wrathall 2012). A much larger evidence base is required if we are to move beyond the normative prescriptions currently circulating of what ought to be done about future environmental migration and begin crafting evidence-based policy that focuses on most likely outcomes and needs.

**Fig. 1:**
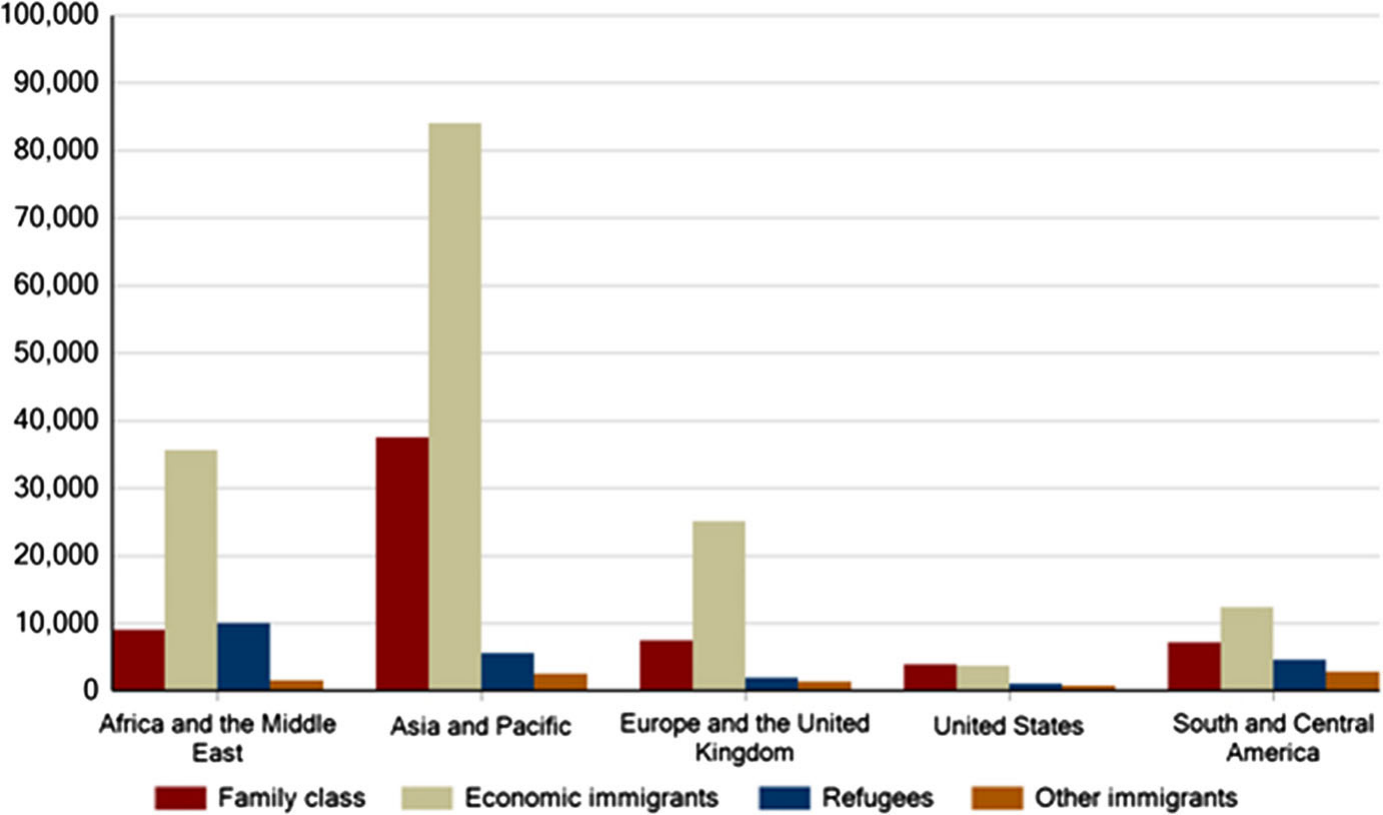
Permanent resident migrants to Canada, by immigration category and source region, 2012. *Source*: http://www.cic.gc.ca/english/resources/statistics/facts2012/permanent/08.asp. Note: It has been official Canadian government policy since the 1990s to set an annual target for legal permanent resident immigrants; this target has typically varied between 200,000 and 300,000 immigrants/year (for the year 2014), it is set for 240,000–265,000 (http://www.cic.gc.ca/english/department/media/notices/2013-11-01.asp)

**Fig. 2:**
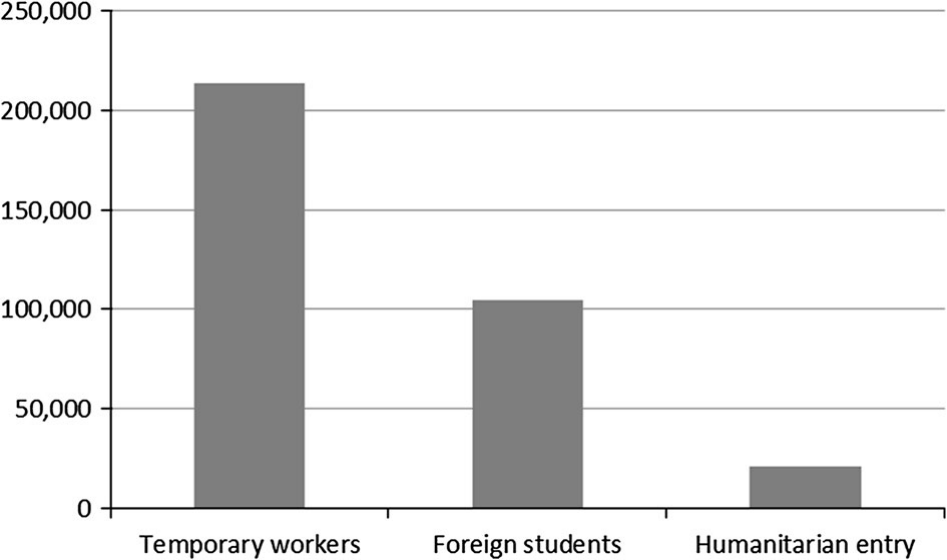
Temporary migrants entering Canada in 2012, by category. *Data source*: http://www.cic.gc.ca/english/resources/statistics/facts2012/temporary/03.asp. Note: People entering Canada as tourist/visitors are not counted. The number of temporary migrants entering Canada has grown steadily over the last decade. The number of temporary foreign workers grew from 102,000 in 2003 to 213,000 in 2012; the number of foreign students grew from 69,000 to 105,000 over the same period (2012 = the most recent year for which statistics are available at time of writing)

In our research, we are looking at this problem from the perspective of international environmental migration to Canada. There are past examples where environmental events overseas, such as earthquakes in Haiti (2010) and Italy (1976), and more recently super typhoons in the Philippines (2013), have prompted the Canadian government to facilitate the movement to Canada of people from affected areas, typically people with pre-established family connections to Canada. Events such as these are, however, exceptional in the broader context of Canadian immigration policy and the movement of migrants to Canada more generally. In 2012, Canada received approximately just over 600,000 legally documented migrants; of these, 257,000 entered to become permanent residents and 350,000 as temporary migrants (Citizenship and Immigration Canada 2012). The majority come from non-contiguous countries in Africa, Asia, the Caribbean, and Latin America (Fig. 1). Those arriving as permanent residents are selected through a variety of programs aimed at facilitating the migration to Canada of skilled labor migrants, international students, business people, and people with family ties to Canada. Temporary migrants consist primarily of labor migrants and international students (Fig. 2); some of these may subsequently apply for permanent status, although this is contingent on a number of regulatory provisions. Canada also offers permanent resident status to convention refugees, some of whom seek protection after making their own way to Canada, while others are assisted in relocating from abroad by relatives, benevolent organizations, or the Canadian government itself.

It is not clear to what extent environmental events and conditions overseas might have an influence, if any, on this broader, heterogeneous mix of migrants to Canada. There is no straightforward or easy way to make such a determination from official records or statistics, primarily because, apart from exceptional cases such as the aforementioned examples of earthquakes and typhoon where the government has created special visa programs, potential migrants are not asked about possible environmental motivations. People who seek to migrate to Canada are required to provide a considerable amount of personal information, such as marital status, language skills, employment history, and personal net worth.^1^ To obtain the necessary visa or permit, they are also typically asked to provide their reasons for seeking entry to Canada; however, these questions are usually tailored to the category in which the potential migrant seeks entry. For example, business immigrants will be asked about their entrepreneurial or investment intentions, family reunification applicants will be asked about their family ties to Canada, and skilled workers will be asked about their qualifications, past work experience, and the types of employment they will seek in Canada and in what cities. Environmental motivations do not figure into any of the categories through which migrants may legally seek entry to Canada, and so such information is rarely sought.

It may be that, given the structure of Canada’s immigration program, people who move internationally for environmental reasons are simply not represented within the flow of migrants to Canada. Alternatively, it may be that environmental factors are a consideration for some groups or individuals seeking entry to Canada, but that they are simply not being asked to identify them. A person for whom environmental factors are a motivating factor for seeking to leave the country of origin could, depending upon his or her family ties or skills set, qualify for legal migration to Canada under one of the various programs without ever being required to disclose that source of motivation. No systematic research has been done previously on this question, so we simply have not known if, let alone how, environmental factors overseas might influence directly or indirectly international migration to Canada. In 2012, we initiated a project to explore this question by working with large immigrant communities in several Canadian cities to find out whether environmental factors in their home countries had any influence on the decisions of their members to migrate to Canada. We began with a participatory research project involving members of several African migrant communities in Ottawa-Gatineau, in which we collaboratively discussed and documented the ways, directly or indirectly, environmental events and conditions in the countries of origin may (or may not) have influenced their migration decisions. Our project has since expanded to working with Haitian, Filipino, and Bangladeshi immigrants and will continue to develop over the next few years. Our key aims are to document whether environmental considerations play a role in migration to Canada and, in situations where they do, identify for which social groups or migrant categories are they most significant, the implications they may have for successful settlement and integration of those migrants, and any other considerations that may be relevant for Canadian migration policymakers. This research briefly reports especially noteworthy findings from the first stage of our project that will be of interest to other scholars in the field.

## Methodology

Given the exploratory nature of our project, we have been using qualitative methods to solicit observations on motivations for migration in terms of the environmental, economic, political, social, and cultural context of their countries of origin. We selected Ottawa-Gatineau for the first stage of our study because we are based here and have existing contacts within its large, diverse migrant population. We began by reaching out to local African communities because that continent is often cited in existing literature as being a significant location of environmentally related migration and, as can be seen from Fig. 1, Africa is a significant source of migrants to Canada across all categories (Morrissey 2014).

Data collection was initiated by first holding open-ended interviews with representatives of African migrant settlement agencies, frontline social workers, and community leaders to explain the objectives of the project, to obtain their initial insights into the broader migration experiences within the communities, and to enlist their assistance in organizing focus groups with members of their respective communities. We fully disclosed the objectives of the project and our intention to ask questions about the possible influence of environment on migrant motivation. We emphasized that we presumed nothing and that it would be a perfectly acceptable research outcome should we be told that environmental factors are of no significance as motivations for migration within their respective communities. This same disclosure and instruction was made at the outset of each of the focus groups we subsequently organized. The focus groups were semi-structured, and a copy of the list of questions used is included in the Supplemental Materials for this research note.

We held seven focus groups consisting of 47 migrants (24 men and 23 women) from the Horn of Africa (4 groups, 28 participants) and from francophone sub-Saharan Africa (3 groups, 19 participants) (Table 1). Participation was entirely voluntary, with participants being recruited by our initial contacts through their respective communities’ social organizations. The only prerequisites for participation beyond willingness were that participants not be minors and that they had migrated to Canada within the last 10 years. Participants received a small honorarium of CAD$40 to compensate them for their time and travel.

**Table 1:** Participants’ countries of birth/origin

Region: Horn of Africa (HA) or sub-Saharan Africa (SSA)	Country of birth/origin	Number of participants (*n* = 47)	% of all participants	Immigration categories (% by country of origin)
HA	Djibouti	8	17	Economic: 2 (25 %)
				Family: 2 (25 %)
				Refugee: 4 (50 %)
HA	Somalia	19	40.4	Family: 4 (21 %)
				Refugee: 13 (68.4 %)
				Other/NA: 2 (10.5 %)
HA	No response	1	2.1	Refugee: 1 (100 %)
SSA	Benin	1	2.1	Family: 1 (100 %)
SSA	Burkina Faso	1	2.1	Family: 1 (100 %)
SSA	Burundi	2	4.2	Refugee: 2 (100 %)
SSA	Cameroon	5	10.6	Economic: 5 (100 %)
SSA	Congo-Brazzaville	1	2.1	Economic: 1 (100 %)
SSA	Cote d’Ivoire	1	2.1	Family: 1 (100 %)
SSA	Democratic Republic of the Congo (DRC)	3	6.4	Economic: 1 (33.3 %)
				Refugee: 2 (66.6 %)
SSA	Guinea	2	4.2	Economic: 2 (100 %)
SSA	Rwanda	2	4.2	Family: 1 (50 %)
				Refugee: 1 (50 %)
SSA	Togo	1	2.1	Economic: 1 (100 %)

Of the groups representing the Horn of Africa, one was a group of francophone migrants from Djibouti and the other three consisted of Somali migrants, mostly refugees. The Somali focus groups were conducted in the Somali language with the help of research assistants; the others were conducted in French. Overall, 23 focus group participants (48.9 %) had come to Canada as refugees (most of them government sponsored), 12 (23.4 %) as skilled workers, 10 (17 %) through family reunification, and two (4.2 %) said “other” or chose not to disclose their immigration category. All participants arrived in Canada as adults. The large majority (41 participants or 87.2 %) had been in the country for 5 years or less, with nineteen of them (40.4 %) being very recent arrivals—less than 2 years—and twenty-two (46.8 %) had been in Canada for a period of between 2 and 5 years. Two participants (4.2 %) had been in Canada for between 5 and 10 years; and four participants (8.5 %) chose not to disclose the date of their arrival. Many participants are highly educated; twenty-eight (59.5 %) have at least a postsecondary degree (Table 2). Eighteen participants (38.3 %) were between ages 18 and 34, twenty-seven (57.4 %) were between ages 35 and 54, one (2.1 %) was 55 or older, and one (2.1 %) did not disclose the information.

**Table 2:** Participants’ levels of education

Levels of education	Number of participants (*n* = 47)	%
Primary school only (less than 8th grade)	5	10.6
Some high school	4	8.5
High school diploma	7	14.9
Some college/specialized/postsecondary/university education	2	4.2
College/specialized/postsecondary/university degree	9	19.1
Completed graduate education	15	31.9
Professional degree, certification, or diploma	4	8.5
Other	1	2.1

Those participants who migrated to Canada as skilled workers said that their main motivation was economic and, to a lesser extent, social. Most of these had previously migrated to Europe (primarily France, Belgium, and Germany) to pursue postsecondary education. Upon completion of their degree, some went back to their country of origin but were unable to find appropriate employment and thus decided to migrate to Canada. Others filed their immigration application directly from Europe. They explained that it was difficult for international students to become permanent residents in Europe (especially in France) and that it was easier for them to immigrate to Canada. In addition, a number of participants believed Canada offered better employment and professional opportunities as well as a better quality of life for their families. For some, Canada’s French–English bilingualism was also an attraction.

The majority of participants who came as refugees were government sponsored^2^; only a few were sponsored by their families, and one participant from Djibouti had arrived independently and asked for asylum. The Somali refugees came to Canada either directly from refugee camps in Somalia or via third countries, including Djibouti, Eritrea, Kenya, South Africa, Syria, and China. While they had little choice in selecting the country of destination, on reaching Canada most decided to settle in Ottawa either because they had preexisting contacts or because they had heard of its well-established Somali community. The other refugees came directly from their countries of origin (e.g., Burundi, Djibouti, DRC, Rwanda). Finally, most of the participants who arrived under the category of family reunification were sponsored by their spouses who had migrated before them; in only one instance was the sponsoring spouse born in Canada.

When asked to reflect upon environmental issues in their countries of origin, a considerable range of environmental challenges was described, which varied from one country to another. When further asked whether these environmental factors had any links directly or indirectly to their decisions to come to Canada, participants’ answers varied, as did the importance they assigned to environmental factors relative to the primary reasons described above. Participants also reflected, often at some length, on how environmental factors influenced internal migration patterns within their home countries. The findings are summarized below.

## Key findings: environmental influences on international migration versus internal migration

Focus group participants generally believe that environmental factors influence population movements within their countries of origin. However, no participants cited environmental factors as having been the immediate or most important reason for their own personal decisions to migrate to Canada. Instead, participants within each group suggested, to the general agreement of others after discussion, that internal migration patterns generated by environmental pressures contributed to larger socioeconomic problems they faced in their home countries and that these in turn *did* influence indirectly their own migration. In other words, their collective view was that environmental factors in their home countries act as second- or third-order contributors to longer, complex chains of interactions that for some groups or individuals can lead to long-distance international migration to Canada.

To begin with, participants were able to describe with considerable detail how environmental problems in their home countries, such as drought and land degradation (Table 3), create ongoing hardship for rural populations and help drive short-distance migration of the rural poor to urban centers. Several participants had directly experienced environmentally related migration within their home countries. A participant from Burkina Faso described how her father, who was a farmer, had in the past regularly migrated to Cote d’Ivoire during times of drought and eventually settled there permanently. Participants from Somalia, most of whom had lived in refugee camps, discussed livelihood strategies they had used in their home country to cope with drought and conflict (which often went hand-in-hand), such as selling off their livestock and participating in rural-to-urban migration. Other focus group participants who had not participated in environmental migration within their home countries described environmental challenges they believed to be linked to internal migration, such as Cameroonian participants who explained how falling water levels in Lake Chad are prompting fishermen to migrate elsewhere. Others described the actual or potential migration effects of floods, landslides, erosion, land degradation, and heat events (Table 3). Somali participants described deforestation in Somalia, how it is driven by the need for wood for cooking and charcoal production, and the competition with pastoralists who scour any remaining vegetation to feed their animals during droughts. Central African participants described deforestation there as being linked with the need for firewood, as well as with mining and commercial logging. Participants from areas where mining is common (e.g., Burundi, Cameroon, Congo-Brazzaville, DRC) reported people being directly displaced by mining companies, with mining-related land degradation and water pollution creating subsequent migration pressures. Participants from Burkina Faso said that export-oriented cotton production in that country contributes to land degradation and rural food insecurity, which in turn prompts internal migration.

**Table 3:** Migration and environmental factors related to human activity, as described by focus group participants

Environmental factors cited by participants	Frequency with which factors were cited		Countries affected	Perceived linkage to migration
	Focus group code (HA = Horn of Africa, SSA = sub-Saharan Africa)	# of times cited		
Drought, desertification, water scarcity	HA#20.2	10	Benin, Burkina Faso, Burundi, Cameroon, Djibouti, Rwanda, Somalia	Somalia: internal rural–rural, rural–urban migration, and migration to Djibouti Burundi to Rwanda (food scarcity) Burkina Faso: cyclical migration to Cote D’Ivoire and Ghana Cameroon: internal rural–rural and rural–urban migration and chain migration rural–urban–regional for fishermen from the Lake Chad region Rwanda: internal cyclical migration
	HA#1.3	4		
	HA#19.3	9		
	HA#27.3	5		
	SSA#12.2	8		
	SSA#19.2	2		
	SSA#26.2	5		
Deforestation	HA#20.2	4	Benin, Burundi, Cameroon, Congo-Brazzaville, Djibouti, DRC, Guinea, Rwanda, Somalia	Mostly internal rural–rural and some rural–urban migration
	HA#1.3	2		
	HA#19.3	1		
	HA#27.3	1		
	SSA#12.2	12		
	SSA#19.2	1		
	SSA#26.2:	3		
Urban ecological decline	HA#20.2	6	Benin, Burkina Faso, Cameroon, Congo-Brazzaville, DRC, Guinea, Rwanda, Togo	Potential factor influencing international migration of urban elites to Europe and Canada
	HA#1.3	0		
	HA#19.3	5		
	HA#27.3	1		
	SSA#12.2	18		
	SSA#19.2	1		
	SSA#26.2	3		
Land degradation other than deforestation (e.g., mining, overly intensive agriculture)	HA#20.2	0	Burkina Faso, Burundi, Cameroon, Congo-Brazzaville, DRC	Mostly internal rural–rural and rural–urban migration, some rural–rural migration between Burundi and Tanzania
	HA#1.3	0		
	HA#19.3	0		
	HA#27.3	0		
	SSA#12.2	5		
	SSA#19.2	4		
	SSA#26.2	2		

Participants were in many instances able to describe the particular patterns of migration that emerge in response to these environmental factors, such as increased participation in rural–urban, rural–rural, and urban–rural migration, depending on the circumstances. In some cases, migration is short-term or temporary; in others, it was described as being seasonal, cyclical, or permanent. For example, a participant from Burkina Faso described how Burkinabe farmers respond to drought with cyclical migrations to Cote d’Ivoire and Ghana, while our Burundian participants explained that rural populations of Burundi cross into Rwanda to escape food scarcity during longer periods of drought. In Rwanda, we were told that farmers follow traditional migration pathways during times of drought, with some seeking to move permanently by buying land in the eastern parts of the country or by crossing into Tanzania. Deforestation, mining, and their associated environmental impacts in Cameroon and DRC contribute to the displacement of mostly rural populations who take refuge in neighboring rural areas.

Participants were particularly knowledgeable about the movement of people between rural and urban areas in their home countries, typically describing it as being highly dynamic. We here again observed the distinctive nature of the Somali experience, as compared with that of other focus groups. Participants from Somalia described how herders who have lost their livestock due to drought often move to urban areas, even though urban food insecurity is already high given the heavy dependence on food produced in nearby rural areas. At the same time, the ongoing conflict in Somalia exacerbates scarcity of food, water, and energy in urban areas, producing a counterflow of urban out-migration to rural areas as urbanites seek to meet their basic needs. In Cameroon, drought sometimes pushes rural populations to migrate to cities, while in DRC, deforestation drives some rural populations to large cities such as Kinshasa. But participants explained that these unskilled rural migrants struggle to make a living in cities, where they must often work in the informal economy, and sometimes end up returning to rural areas.

Participants from both the Horn of Africa and sub-Saharan countries discussed at length how urban ecological quality is declining in the source countries due to rapid, unplanned urban growth, and related challenges such as lack of housing, inadequate waste management infrastructure, growing air and water pollution, and in some cases, declining groundwater and disease outbreaks like cholera (Table 4). Participants see environmentally related out-migration from rural areas to cities as being an important contributor to this overall trend toward urban ecological decline. Participants also expressed concern that high rates of urban unemployment generate a degree of urban social unrest. Unskilled rural migrants were described as struggling with high unemployment in most of the source countries represented; participants also expressed concerns about educated urban youth who struggle to find work. All participants described the cities in their countries of origin as being relatively unhealthy, ecologically degraded, and potentially unsafe, and the combined effects provide a general stimulus to prompt educated and skilled urbanites such as themselves to seek professional opportunities and a better quality of life abroad.

**Table 4:** Problems associated with rapid urban growth, as described by focus group participants

Environmental factor	Countries	Description and related impacts
Waste management	Benin, Cameroon, DRC, Guinea, Rwanda, Somalia, Togo	Lack of infrastructure, planning, no recycling, and poor waste management
Sanitation problems	Cameroon, Congo-Brazzaville, Djibouti, DRC, Guinea, Somalia, Togo	Lack of infrastructure, planning, and waste management services. Contaminated water leads to sanitation issues, waterborne illnesses (cholera)
Air pollution	Benin, Burkina Faso, Cameroon, DRC, Rwanda, Togo	Lack of regulation of industry and transportation causes air pollution; cities covered by smoke and smog; dust in the air leads to respiratory problems
Water contamination and lack of safe fresh water	Benin, Cameroon, Congo-Brazzaville, Djibouti, DRC, Guinea, Somalia	Industrial waste and human waste are dumped into urban waterways. Fisheries are polluted, affecting livelihoods
Flooding	Burkina Faso, Cameroon, Congo-Brazzaville, Djibouti, DRC, Somalia, Togo	Poor infrastructure leads to floods, ponding during heavy rains, in turn results in sanitation problems, waterborne illnesses (cholera), erosion, and damage to buildings
Rising temperatures	Benin, Cameroon, Djibouti, DRC, Somalia	Loss of vegetation and green spaces exacerbates urban heat island effect
Overcrowding and shantytowns/unplanned urban growth	Benin, Djibouti, DRC, Somalia	Contribute to erosion; sensitive to flooding and landslides; put stress on local resources (electricity, water); unsustainable living conditions; insecurity; and lack of personal safety

## Common themes meriting further research

Despite the inherently qualitative nature of the focus group discussions and the subjective, personal experiences reflected therein, some common themes arose. A first is that environmental factors in the home country are not a proximate cause or influence on participants’ decisions to migrate to Canada. Participants also did not know of other members of their communities in Ottawa-Gatineau having been motivated principally by environmental reasons to come to Canada. This is despite the fact that all focus group participants were able to list with ease a variety of environmental challenges faced in their home countries, could identify with some precision the types of people most affected by such challenges, and could describe how those environmental challenges affected population movements within their home countries or to contiguous ones. Participants’ descriptions of environment migration dynamics in Africa are often consistent with empirical evidence in the many case studies reviewed by Morrissey (2014). However, it is clear that the people who become environmental migrants within Africa are generally not present in the stream of migrants coming to Ottawa-Gatineau from the African source countries represented here. The possible exception is that of Somalia, but even in that case, participants who had personal experience with environmental hardship and/or environmental migration in that country cited personal security, economic considerations, and family reunification as being their foremost motivations for migrating to Canada.

This begs the question: If environmental migration is taking place in the source countries represented, why are environmental migrants not represented in international migration from those countries to the Ottawa-Gatineau region? Here, with the help of focus group participants, we can offer some suggestions. A starting point is the structure of Canada’s immigration program, which facilitates the movement of skilled workers (where the skills require considerable investment in formal education and training) and people with family connections to Canada. Would-be migrants without a Canadian family member to sponsor them must be of independent financial means. The types of people most disproportionately affected by environmental changes described in the focus groups—agricultural workers, fishers, laborers in primary resource sectors, and so forth—would not qualify for migration to Canada in the absence of a family sponsorship.

The experiences of participants from Somalia, most of whom sought permanent residence as refugees, differed from participants from other groups, but even here our Somali participants typically had Canadian relatives or connections to the Somali-Canadian community prior to migrating to Canada. It is likely not coincidental that participants in the Somali focus group were much more likely to have had a personal, firsthand experience with environmental hardship and/or environmentally related migration as compared with participants in the other focus groups, who were primarily skilled migrants from less conflict-torn urban centers. This suggests our future research must examine more closely the experiences of people coming to Canada within the refugee movement.

**Fig. 3:**
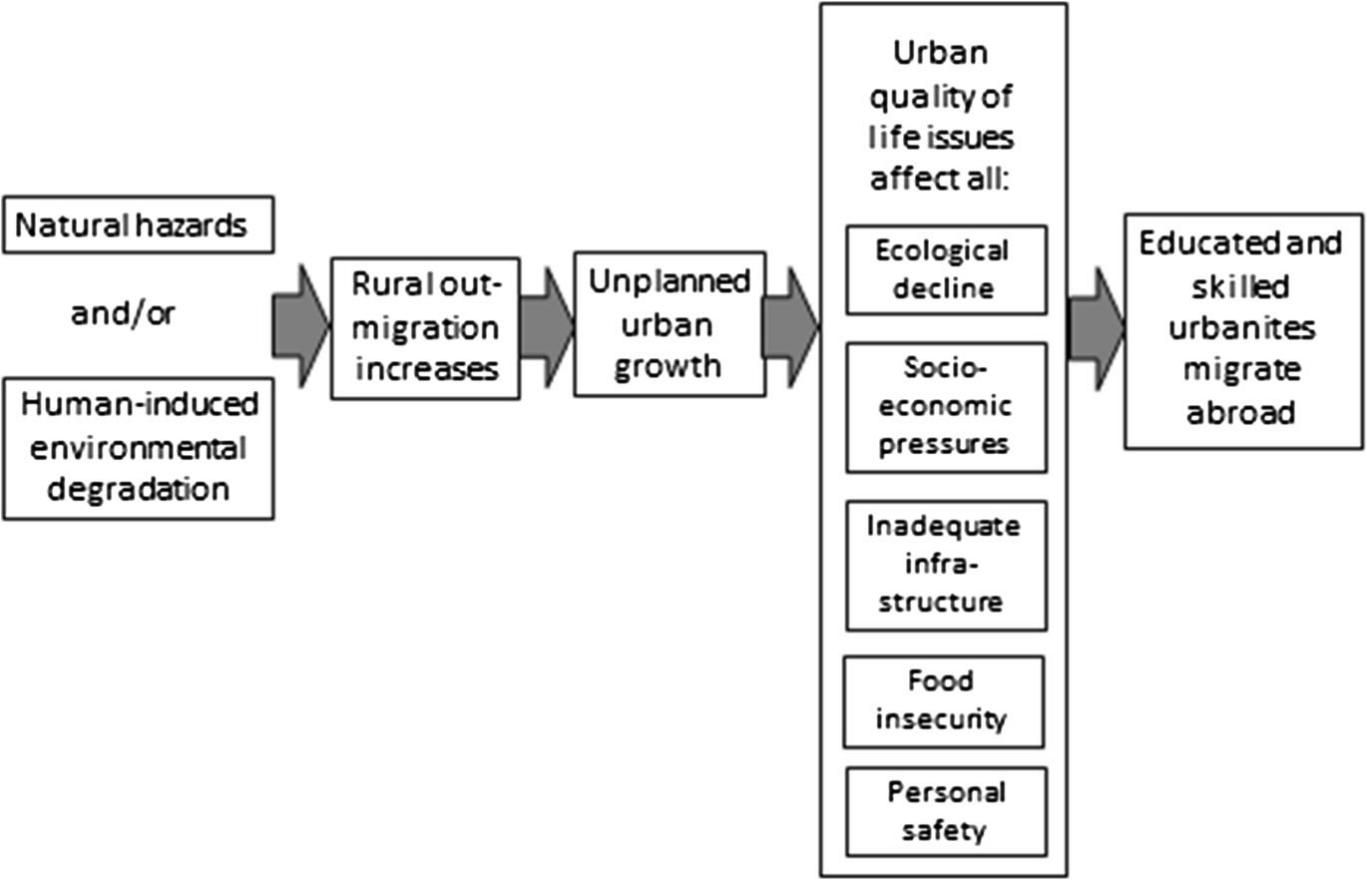
Influence of environmental factors in source countries on international migration causality, as described by focus group participants

This does not mean that environmental factors have had no influence whatsoever on the circumstances that have led non-Somali groups to migrate to Canada. Participants suggest there is an indirect connection between internal migration processes in these source countries and international migration from them. Within the source countries, seasonal dryness and drought combine with ecological decline resulting from human activities to become important drivers of internal population flows between rural and urban areas. The consequent influx of rural migrants into urban centers contributes to urban ecological decline, food insecurity, and socioeconomic instability. According to our participants, these latter phenomena become additional considerations for educated and skilled middle-class urbanites who might already be contemplating migration to an international destination like Canada for economic or family reasons. This suggests there is a quality-of-life consideration being made in addition to the income differentials between migration source countries and potential destinations that have been suggested elsewhere as being key drivers of international environmental migration from less developed countries (Lilleør and van den Broeck 2011). The overall dynamic described in our focus groups is shown in simplified fashion in Fig. 3.

For Somali participants, the linkages between environment, conflict, and insecure access to food and basic resources are much more pronounced than participants from the other countries. People in Somalia who suffer from these ongoing problems simply do not have access to Canada’s immigration system unless they are fortunate enough to have a close family member or other potential sponsor already established there. This structural barrier is not unique to Somalia, however. The people described in our focus groups as being most directly susceptible to environmental displacement in African countries—typically the poor, rural, and/or marginalized—would not qualify for independent admission to Canada. Their limited financial resources limit their migration possibilities to internal and/or intra-regional destinations. By contrast, members of the educated urban elite who are not directly affected by environmental factors do have the ability to seek admission to Canada, should they so choose. Those represented in our focus groups suggest that rapidly growing urban populations and declining urban ecological conditions—which they (rightly or wrongly) ascribe at least in part to influxes of internal environmental migrants—are a consideration for the would-be international migrant and are likely to grow in significance. This subject stands out as warranting greater research attention given its relative absence in the empirical literature and given the scale of the urban ecological and demographic challenges facing African cities in coming years described by African researchers such as Crush and Frayne (2011).

Our participants held various views on the relative significance of environmental factors versus social, economic, and political ones in shaping migration. Some saw environmental factors as central to the challenges that populations in their countries of origin face and thus to migration. More participants, however, suggested that political instability, weak governance, endemic poverty, lack of economic alternatives, and lack of infrastructure were at the root of environmental problems in the first place—and so these were seen as being the main drivers of migration. In all cases, participants clearly see the environment not as an independent or exogenous force, but as being intertwined with non-environmental factors in migration causality. Our findings support the continued development of approaches that treat environmental migration as an outcome of dynamic interactions between human and environmental systems (as in Black et al. 2011). In particular, our findings point to the need to examine more carefully how structural barriers in recipient countries of international migration, such as Canada, constrain the adaptive migration options of those most greatly exposed to environmental risks. In short, there remains much to learn about environmental influences on South–North migration patterns, reinforcing the need for considerably more empirical research on the subject.

## Electronic supplementary material

Below is the link to the electronic supplementary material.

Supplementary material 1 (DOC 48 kb)

## Footnotes

1 Further details about Canada’s immigration program are found at www.cic.gc.ca. In interests of full disclosure, the second author was a Canadian immigration/overseas visa officer for much of the period 1990–2002.

2 Canadian permanent residents, citizens, and benevolent organizations (e.g., church groups) may offer to sponsor a refugee living abroad to be resettled in Canada; the refugee being sponsored is typically known to the sponsor, who undertakes to provide resettlement assistance to the refugee. The government of Canada also of its own accord resettles a certain number of UN Convention Refugees from abroad each year and provides resettlement assistance directly. This latter group is known as government-assisted refugees.

## References

[CR1] Afifi T (2011). Economic or environmental migration? The push factors in Niger. International Migration.

[CR2] Alscher S (2011). Environmental degradation and migration on Hispaniola Island. International Migration.

[CR3] Barbier B, Yacouba H, Karambiri H, Zoromé M, Somé B (2009). Human vulnerability to climate variability in the Sahel: Farmers’ adaptation strategies in northern Burkina Faso. Environmental Management.

[CR4] Bates DC (2002). Environmental refugees? Classifying human migrations caused by environmental change. Population and Environment.

[CR5] Biermann F, Boas I, Scheffran J, Brzoska M, Brauch HG, Link PM, Schilling J (2012). Climate change and human migration: Towards a global governance system to protect climate refugees. Climate change, human security and violent conflict.

[CR6] Black R, Adger WN, Arnell NW, Dercon S, Geddes A, Thomas D (2011). The effect of environmental change on human migration. Global Environmental Change.

[CR7] Citizenship and Immigration Canada. (2012). Facts and figures 2012—Immigration overview: Permanent and temporary residents. http://www.cic.gc.ca. Cited August 26, 2013.

[CR8] Crush JS, Frayne GB (2011). Urban food insecurity and the new international food security agenda. Development Southern Africa.

[CR9] Dun O (2011). Migration and displacement triggered by floods in the Mekong Delta. International Migration.

[CR10] El-Hinnawi E (1985). Environmental refugees.

[CR11] Findley SE (1994). Does drought increase migration? A study of migration from rural Mali during the 1983–1985 drought. International Migration Review.

[CR12] Foresight. (2011). *Migration and global environmental change*. Final Project Report. London. http://www.bis.gov.uk/assets/bispartners/foresight/docs/migration/11-1116-migration-and-global-environmental-change.pdf.

[CR13] Füssel H-M (2010). How inequitable is the global distribution of responsibility, capability, and vulnerability to climate change: A comprehensive indicator-based assessment. Global Environmental Change.

[CR14] Fussell E, Sastry N, VanLandingham M (2010). Race, socioeconomic status, and return migration to New Orleans after Hurricane Katrina. Population and Environment.

[CR15] Gibb C, Ford J (2012). Should the United Nations Framework Convention on Climate Change recognize climate migrants?. Environmental Research Letters.

[CR16] Gill N (2010). “Environmental refugees”: Key debates and the contributions of geographers. Geography Compass.

[CR17] Gray CL (2009). Environment, land, and rural out-migration in the southern Ecuadorian Andes. World Development.

[CR18] Gray CL (2010). Gender, natural capital, and migration in the southern Ecuadorian Andes. Environment and Planning A.

[CR19] Gray C, Mueller V (2012). Drought and population mobility in rural Ethiopia. World Development.

[CR20] Gray C, Mueller V (2012). Natural disasters and population mobility in Bangladesh. Proceedings of the National Academy of Science.

[CR21] Groen J, Polivka A (2010). Going home after Hurricane Katrina: Determinants of return migration and changes in affected areas. Demography.

[CR22] Gutmann MP, Field V (2010). Katrina in historical context: Environment and migration in the U.S. Population and Environment.

[CR23] Henry S, Piché V, Ouédraogo D, Lambin EF (2004). Descriptive analysis of the individual migratory pathways according to environmental typologies. Population and Environment.

[CR24] Hunter LM (2005). Migration and environmental hazards. Population and Environment.

[CR25] Lilleør HB, Van den Broeck K (2011). Economic drivers of migration and climate change in LDCs. Global Environmental Change.

[CR26] Marchiori L, Schumacher I (2011). When nature rebels: International migration, climate change, and inequality. Journal of Population Economics.

[CR27] McLeman R, Barrett C (2013). Labor migration and food security in a changing climate. Food security and sociopolitical stability.

[CR28] McLeman R, Smit B (2006). Migration as an adaptation to climate change. Climatic Change.

[CR29] Morrissey J, Piguet E, Laczcko F (2014). Environmental change and human migration in Sub-Saharan Africa. People on the move in a changing climate.

[CR30] Myers N (2002). Environmental refugees: A growing phenomenon of the 21st century. Philosophical Transactions of the Royal Society London: Biological sciences: Series B.

[CR31] Nawrotzki RJ, Riosmena F, Hunter LM (2013). Do rainfall deficits predict U.S.-bound migration from rural Mexico? Evidence from the Mexican census. Population Research and Policy Review.

[CR32] Rappaport J (2007). Moving to nice weather. Regional Science and Urban Economics.

[CR33] Schultz J, Elliott JR (2013). Natural disasters and local demographic change in the United States. Population and Environment.

[CR34] Shrestha S, Bhandari P (2007). Environmental security and labor migration in Nepal. Population and Environment.

[CR35] Tacoli C (2009). Crisis or adaptation? Migration and climate change in a context of high mobility. Environment and Urbanization.

[CR36] Wrathall DJ (2012). Migration amidst social-ecological regime shift: The search for stability in Garifuna villages of northern Honduras. Human Ecology.

