# Supplementary material for: Environmental influences on African migration to Canada: focus group findings from Ottawa-Gatineau
Source: Popul Environ. 2014 Jul 12;36(2):234–51. doi: 10.1007/s11111-014-0214-3 (PMC4223532; doi:10.1007/s11111-014-0214-3)
Supplement: Supplementary file 1 — Supplementary material 1 (DOC 48 kb) [file 11111_2014_214_MOESM1_ESM.doc]

**Focus group interview guide**

The questions in this interview guide will be used in focus groups with individuals constitutive of our particular research groups – mainly immigrant communities from various selected world regions (Central Africa, Horn of Africa, Central America) and countries (Haiti, Dominican Republic, Philippines) among others. The objective of the focus groups will be to investigate whether environmental conditions played a role, directly and/or indirectly, in their decision to migrate to Canada, the role of environmental factors in shaping their migration strategies and experiences, as well as any lasting effects of environmental factors on their settlement process and needs.

| **Theme: Introduction to the research project** | |
| --- | --- |
| **Environment and international migration to Canada** | - A number of international studies have suggested that droughts, floods, land degradation, tropical cyclones and other environmental hazards are affecting migration patterns in many parts of the world already, and are expected to increase in coming decades as a result of climate change. - Canada is one of the leading destinations for international migrants and a country with many environmental amenities. Canadians might therefore ask if environmental conditions in key immigrant source countries are influencing people’s decisions to come to Canada. If so, how? If environment does matter for some immigrant groups, does it create special considerations to ensure immigrants integrate successfully into Canadian society? - XXX of the XXX University are leading the first-ever study to determine how environmental factors in other countries influence migration to Canada and, if so, how environmental factors may affect the settlement and incorporation prospects of migrants once they arrive. - Introductions: we would like to invite each of you to introduce yourselves, and share with others why you were interested in participating in this Focus Group. |
| **Theme: Environmental factors and migration decisions** | |
| **Environmental factors** | We would like to discuss the potential role of environmental factors in your decisions to leave your country of origin.   - Based on your experience, did any environmental factors influence your decision to leave your country of origin? - If so, what types of environmental factors affected your migration decisions? - What do we mean by environmental factors? (e.g., drought, floods, air pollution, natural disasters, etc.) How do you understand the notion of “environment”? - How do these compare with non-environmental factors (e.g., economic, political, social, cultural, other) in terms of significance? Can you explain? - To what extent did environmental factors combine (or not) with non-environmental factors? - Can you describe your town or city of origin? - And how has it been or is still being affected by environmental conditions? - Are you aware of other environmental factors that affect your country or region of origin? - Do these cause displacement or migration within or outside your country/region of origin? |
|  | Before moving on, are there any additional thoughts you wish to share around these issues? |
| **Migrants’ strategies of immigration** | We are now interested in hearing about your migration experiences to Canada.   - When was the first time you, or another member of the family, wished to migrate? - Based on which criteria did you make the final decision to leave your country of origin and to live abroad? - Is the environment one of your criteria? And why? - How did you obtain information about migrating to Canada? What kinds of information did you receive? - Did you migrate to Canada alone or with family members? - Did you directly come to Canada from your city/town of origin? - If not, to which city or country did you migrate to before moving to Canada? - How was your migration experience? - Based on your knowledge, is your experience/venture common? - Did you face any challenges? - Based on your contacts, are there people who would like to immigrate to Canada but cannot? - If so, what types of barriers stopped or are stopping them (e.g., women; children; those who are poor, have low levels of education, no knowledge of Canada’s official languages; those with health problems; etc.)? |
|  | Before moving on, are there any additional thoughts you wish to share around these issues? |
| **Theme: Environmental factors and settlement** | |
| **Settlement destination** | We now want to ask you about how environmental factors may have shaped your selection of a destination.   1. Canada as a country of settlement:  - Did Canada’s environmental characteristics/qualities play a role in your decision to come here? - If so, which ones? (e.g., climate, green spaces/nature, air quality, availability of resources such as safe water, etc.) - How did you know or find out about Canada’s environmental conditions? - Were there any non-environmental factors also at play? - If so, which ones? (e.g., economy, politics, culture, multiculturalism, established community, existing networks, etc.) - What was the significance of environmental factors in relation to non-environmental factors?  1. Ottawa-Gatineau or Toronto as destination cities:  - Within Canada, how did Ottawa-Gatineau or Toronto became your living city? - Did environmental factors play a role? - If so, which ones? (e.g., city size, green spaces/presence of nature, climate, location, air quality, etc.) - How did you know or find out about the city’s environmental conditions? - Did you settle in other cities/provinces before coming here? - Explain why you might have relocated within Canada. - Were there any non-environmental factors that also influenced your settlement decisions? - If so, which ones? (e.g., culture, language, presence of an established community, existing networks, availability of social and health services, employment, education, politics, public transit, housing, etc.) - What was the significance of environmental factors in relation to non-environmental factors? |
|  | Before we close, are there any additional thoughts you wish to share around these issues? |
| **Theme: Environmental factors and settlement needs** | |
| **Settlement needs and integration process** | Our last set of questions is regarding the role of environmental factors in shaping migrants’ settlement needs and integration process.   - What were your needs upon arrival in Canada? - Were these related to any environmental factors that you experienced in your country of origin? If so, can you explain? - Were there any particular needs that were not met? - If so, can you explain? - How did you hear about newcomer services? Did you receive sufficient information? - What were your main concerns or priorities once you arrived to Canada? (e.g., stable legal status in Canada; health issues; family reunification; employment, education; going back to the country of origin; solidarity with the people in the home country; other) - Did these change over time? - What were the main challenges you faced after the initial settlement process in Canada? - Based on your contacts, are they common? Please, explain. |
|  | Before we close, are there any additional thoughts you wish to share around these issues?  We thank you for your time and participation. |
